# Lack of transforming growth factor-β signaling promotes collective cancer cell invasion through tumor-stromal crosstalk

**DOI:** 10.1186/bcr3217

**Published:** 2012-07-02

**Authors:** Lauren A Matise, Trenis D Palmer, William J Ashby, Abudi Nashabi, Anna Chytil, Mary Aakre, Michael W Pickup, Agnieszka E Gorska, Andries Zijlstra, Harold L Moses

**Affiliations:** 1 Department of Cancer Biology, Vanderbilt-Ingram Cancer Center, Vanderbilt University School of Medicine, 2220 Pierce Avenue, 771 Preston Research Building, Nashville, TN 37232, USA; 2 Department of Pathology, Microbiology, and Immunology, Vanderbilt University School of Medicine, 1161 21st Avenue South, C-2314 Medical Center North, Nashville, TN 37232, USA; 3 Department of Medicine, Vanderbilt University School of Medicine, 1161 21st Avenue South, D-3100 Medical Center North, Nashville, TN 37232, USA

## Abstract

**Introduction:**

Transforming growth factor beta (TGF-β) has a dual role during tumor progression, initially as a suppressor and then as a promoter. Epithelial TGF-β signaling regulates fibroblast recruitment and activation. Concurrently, TGF-β signaling in stromal fibroblasts suppresses tumorigenesis in adjacent epithelia, while its ablation potentiates tumor formation. Much is known about the contribution of TGF-β signaling to tumorigenesis, yet the role of TGF-β in epithelial-stromal migration during tumor progression is poorly understood. We hypothesize that TGF-β is a critical regulator of tumor-stromal interactions that promote mammary tumor cell migration and invasion.

**Methods:**

Fluorescently labeled murine mammary carcinoma cells, isolated from either MMTV-PyVmT transforming growth factor-beta receptor II knockout (TβRII KO) or TβRII^fl/fl ^control mice, were combined with mammary fibroblasts and xenografted onto the chicken embryo chorioallantoic membrane. These combinatorial xenografts were used as a model to study epithelial-stromal crosstalk. Intravital imaging of migration was monitored *ex ovo*, and metastasis was investigated *in ovo*. Epithelial RNA from *in ovo *tumors was isolated by laser capture microdissection and analyzed to identify gene expression changes in response to TGF-β signaling loss.

**Results:**

Intravital microscopy of xenografts revealed that mammary fibroblasts promoted two migratory phenotypes dependent on epithelial TGF-β signaling: single cell/strand migration or collective migration. At epithelial-stromal boundaries, single cell/strand migration of TβRII^fl/fl ^carcinoma cells was characterized by expression of α-smooth muscle actin and vimentin, while collective migration of TβRII KO carcinoma cells was identified by E-cadherin^+^/p120^+^/β-catenin^+ ^clusters. TβRII KO tumors also exhibited a twofold greater metastasis than TβRII^fl/fl ^tumors, attributed to enhanced extravasation ability. In TβRII KO tumor epithelium compared with TβRII^fl/fl ^epithelium, Igfbp4 and Tspan13 expression was upregulated while Col1α2, Bmp7, Gng11, Vcan, Tmeff1, and Dsc2 expression was downregulated. Immunoblotting and quantitative PCR analyses on cultured cells validated these targets and correlated Tmeff1 expression with disease progression of TGF-β-insensitive mammary cancer.

**Conclusion:**

Fibroblast-stimulated carcinoma cells utilize TGF-β signaling to drive single cell/strand migration but migrate collectively in the absence of TGF-β signaling. These migration patterns involve the signaling regulation of several epithelial-to-mesenchymal transition pathways. Our findings concerning TGF-β signaling in epithelial-stromal interactions are important in identifying migratory mechanisms that can be targeted as recourse for breast cancer treatment.

## Introduction

Transforming growth factor beta (TGF-β) is a pleiotropic cytokine that regulates growth arrest, cell motility, development, and differentiation [1-4]. TGF-β signaling is also instrumental in the tumor microenvironment by influencing both tumor development and metastasis [4], and it is frequently dysregulated in breast cancers [5-7]. In the mammary epithelium, attenuation of TGF-β signaling using a dominant negative type II transforming growth factor-beta receptor (TβRII) resulted in lobular alveolar hyperplasia and an increased rate of tumor formation in conjunction with a *TGF-α *transgene [8]; however, decreased pulmonary metastasis resulted when dominant negative TβRII was expressed along with a *c-Neu *transgene [8,9]. Conversely, activation or overexpression of TGF-β signaling in mammary carcinoma cells expressing either the *c-Neu *transgene or the polyoma virus middle T antigen (PyVmT) transgene delayed tumor onset but enhanced pulmonary metastasis [9-11]. Taken together, these observations suggest a tumor-suppressive role of TGF-β during tumor initiation and early tumor progression, while additionally implicating TGF-β in promotion of late-stage tumorigenesis. Mammary-specific ablation of TβRII also supported the role of TGF-β as a tumor suppressor but challenged the dogma of TGF-β as a metastatic promoter. Conditional knockout of TβRII in mammary epithelial cells expressing PyVmT led to decreased tumor latency; however, in contrast to attenuated TGF-β signaling models, TβRII ablation increased pulmonary metastasis [12,13].

This dual role of TGF-β as both tumor suppressor and promoter has therefore presented a dichotomy in which TGF-β signaling is context dependent and cancer type dependent. Consequently, epithelial-autonomous TGF-β signaling cannot solely be responsible for influencing tumor behavior. The tumor microenvironment, an abundant source of TGF-β [4], is comprised of diverse cell populations, such as epithelial, stromal, vascular, and immune cells, working coordinately to promote tumor progression. Epithelial-stromal crosstalk in tumorigenesis has garnered much attention. It has been shown that epithelial TGF-β signaling regulates fibroblast recruitment and activation [4,14]. Concurrently, stromal TGF-β signaling suppresses tumorigenesis in adjacent epithelia while its ablation potentiates tumor formation [15,16]. Fibroblasts can also lead carcinoma cells along self-generated extracellular matrix tracks during carcinoma cell migration and invasion [17]. Transient TGF-β signaling in these invading cells can induce single motility, permitting hematogeneous and lymphatic invasion [18,19]. In contrast, lack of active TGF-β signaling results in collective invasion and lymphatic spread [18]. This illustrates the important role of carcinoma cell TGF-β signaling in determining the mode of cell migration and invasion.

The adaptability of invading cells is evident in multiple forms of cell migration. Single cells invade in either an amoeboid or mesenchymal manner characterized by non-epithelial morphology, loss of cell-cell contacts, and presence of actin stress fibers [20]. Whereas amoeboid cells move through matrix pores, mesenchymal migration additionally employs proteolytic remodeling of the extracellular matrix. Collective invasion also relies on local remodeling of the extracellular matrix [21] and occurs by two-dimensional sheet migration or three-dimensional group or strand migration [22]. These cellular cohorts are heterogeneous, comprised of leading and following cells. Leading cells, which may exemplify mesenchymal properties, survey microenvironmental surroundings, relay extrinsic guidance cues to following cells, and forge clustered migration [23]. Amoeboid, mesenchymal-like, and collective cell migration have all been identified in breast cancer [24]. Inflammatory breast cancer, associated with high rates of metastasis and mortality, is marked by evidence of tumor emboli or clusters that maintain p120 and E-cadherin expression through translational control [25]. Collective clusters are also characteristic of invasive ductal carcinoma [26]. On the contrary, lobular carcinoma frequently manifests single cell or strand migration [3,27].

TGF-β potently stimulates cellular migration and invasion of fibroblasts and epithelial cells by promoting fibroblast transdifferentiation into invasive myofibroblasts and by driving an epithelial-to-mesenchymal transition (EMT) frequently associated with invasive tumors [3,28-30]. These observations support the hypothesis that TGF-β regulates migration patterning through tumor microenvironmental interactions, such as epithelial-stromal crosstalk. These spatially, temporally, and biologically complex interactions can make *in vivo *TGF-β signaling studies difficult. We therefore chose to study epithelial-stromal crosstalk through an integrated systems analysis, combining genetically engineered mouse models and the use of the chicken embryo chorioallantoic membrane (CAM) model [31]. Mammary tumor cells xenografted onto the CAM thrive in large part due to robust vascularization of the nascent tumor in the CAM. The CAM model also offers several advantages over other model systems. First, the *ex ovo *model affords long-term intravital imaging for up to 72 hours of continual imaging. Second, this model system enables real-time tracking of cellular behavior throughout the embryo lifespan, allowing for multiple imaging timepoints without compromising host viability. Lastly, in both the *ex ovo *and *in ovo *models, the chicken embryo presents minimal xenograft rejection since the embryo maintains immature, maternal B-cell populations incapable of full immune activity [32,33].

Using both the *ex ovo *and *in ovo *CAM models, we characterized how tumor cell migration and invasion utilizes TGF-β-mediated epithelial-stromal interactions. We found that mammary fibroblasts enhance the migratory potential of carcinoma cells in either a single cell/strand migration when epithelial TGF-β signaling is present or in a collective migration in its absence. Furthermore, the collective migration and invasion observed correlated with increased metastasis. Our data demonstrate that carcinoma cell TGF-β signaling regulates migration patterning, metastasis, and junctional protein expression at the invasive tumor front. The data also implicate a TGF-β-mediated cell-autonomous migratory behavior evident only during stromal influence on epithelial cells.

## Materials and methods

### Cell lines, transfection, and treatment

Mammary tumor epithelial cells - isolated from either mouse mammary tumor virus (MMTV)-PyVmT;MMTV-Cre;TβRII^fl/fl ^(transforming growth factor-beta receptor II knockout (TβRII KO)) mice or MMTV-PyVmT;TβRII^fl/fl ^(control) mice [12] - and Fsp-Cre;TβRII^fl/fl ^(partial TβRII KO) fibroblasts [15] were used in xenografts for *ex ovo *and *in ovo *CAM assays. Both types of epithelial cells were transduced with lentiviral enhanced GFP (kind gift from the Pietenpol Laboratory, Vanderbilt University, Nashville, TN, USA) for intravital imaging. Fibroblasts were labeled with a cell permeable dye (DiIC_18_(5)-DS; Molecular Probes™, Eugene, OR, USA). For all cell combination experiments, fibroblasts were used at a 2.5:1 ratio to promote the most aggressive behavior of epithelial cells (data not shown). A human TβRII retroviral construct (plasmid 19147; Addgene, Cambridge, MA, USA) was used for reconstitution of TGF-β signaling in TβRII KO epithelia. Phoenix packaging cells were transfected with 8 μg construct for 6 hours, followed by 48-hour viral production. TβRII KO epithelia were then infected for 6 hours and subsequently maintained with 1 μg/ml puromycin for selection. Additionally, any TGF-β treatment of cell lines was completed using 1 ng/ml TGF-β1 (R&D Systems, Minneapolis, MN, USA) for 2.5 hours prior to RNA or protein collection.

### *Ex ovo *chorioallantoic membrane assay

Chicken embryos were placed into sterile weigh boats with plastic lids at day 4 post incubation. On day 10 post incubation, enhanced GFP-expressing breast epithelial cells alone or in combination with fibroblasts were grafted onto the CAM. Intravital imaging began on day 12 post incubation. Fully automated upright fluorescent microscopes (Olympus BX61 WI and BX60 M; Olympus America, Inc., Center Valley, PA, USA) were used for imaging fluorescent cells. Time-lapse images were captured every 15 minutes for the duration of the experiment. Analysis of cell velocity, migration distance, and digital processing was achieved through Volocity^® ^software (Improvision, PerkinElmer, Inc., Waltham, MA, USA) using protocols described previously [31]. Two-photon microscopy of CAM tumors was subsequently completed (Vanderbilt Cell Imaging Shared Resource, Nashville, TN, USA). Embryonated eggs for all chicken CAM assays were graciously provided by the Tyson Food Corporation (Springdale, AR, USA).

### *In ovo *chorioallantoic membrane assay

The CAM was prepared as described previously [34]. Briefly, the CAM was dropped from the eggshell on day 10 post incubation. At this time, mammary epithelial cells alone or in combination with fibroblasts were grafted onto the CAM. Tumor-bearing animals were sacrificed and tumor tissue and distant CAM were collected 7 to 10 days post grafting. Distant CAM was classified as any part of the CAM in which the primary tumor was not grafted. In this way, any piece of distant CAM is a metastatic site. To collect distant CAM at the time of sacrifice, the eggshell was cut radially into two equivalent halves. Two circular areas of CAM, identical in size, were harvested from each eggshell half using a boring tool. The resulting four pieces of CAM were then analyzed via murine *Alu *PCR for the presence of disseminated cells.

### Murine *Alu *PCR

To quantify metastatic cell dissemination in the CAM, the CAM DNA was first extracted using the SYBR^® ^Green Extract-N-Amp Tissue PCR Kit (Sigma^®^, St. Louis, MO, USA). DNA was then analyzed through the use of quantitative murine *Alu *PCR (forward primer, 5'-GGGCTGGTGAGATGGCTCAGTGG-3'; reverse primer, 5'-CTTCAGACACACCAGAAGAGGG-3') [35]. Cycle threshold values were subjected to statistical analyses after normalization to chicken glyceraldehyde-3-phosphate dehydrogenase (forward primer, 5'-GAGGAAAGGTCGCCTGGTGGATCG-3'; reverse primer, 5'-GGTGAGGACAAGCAGTGAGGAACG-3').

### *In ovo *experimental metastasis assay

Injections were performed as previously described [34]. In brief, fluorescently labeled carcinoma cells alone or in combination with fibroblasts were injected intravenously into the allantoic vein of the embryo on day 12 post incubation. Initial cell arrest was assessed at 6 hours, and subsequent extravasation and proliferative capability was assessed at 18 and 24 hours (72 hours was used as an additional timepoint). At these timepoints, cell dissemination was analyzed as described above (see *In ovo *chorioallantoic membrane assay). To label the host chicken vasculature, embryos were injected intravenously with 100 μl of 500 μg/ml rhodamine *Lens culinaris *agglutinin (Vector Laboratories, Burlingame, CA, USA) into the allantoic vein. Imaging of epithelial cells and host vasculature was completed using a fully automated upright fluorescent microscope (Olympus BX61 WI). Digital processing was achieved through Volocity^® ^software (Improvision).

### Laser capture microdissection and expression analysis

Laser capture microdissection (LCM) was performed on 5 μm frozen *in ovo *tumor sections on an Arcturus PixCell IIe microscope (Molecular Devices, Sunnyvale, CA, USA) at the Vanderbilt Translational Pathology Shared Resource (Nashville, TN, USA). LCM-captured RNA was isolated using an RNAqueous-Micro kit (Ambion, Austin, TX, USA) and validated for array quality (Vanderbilt Genome Sciences Resource). Subsequent cDNA synthesis and amplification was completed using a RT^2 ^Nano PreAMP cDNA Synthesis Kit (SA Biosciences™, Frederick, MD, USA). Samples, three control tumors and three KO tumors, were individually assayed on EMT RT^2 ^Profiler™ quantitative PCR arrays (SA Biosciences™) in a Bio-Rad iCycler (Hercules, CA, USA). Analysis was completed using web-based RT^2 ^Profiler™ PCR array data analysis (SA Biosciences™). Selected gene targets were either 10-fold or greater upregulated or downregulated when comparing our TβRII KO tumors with our TβRII^fl/fl ^tumors.

### Expression analysis

Total cell RNA was collected using TRIzol (Invitrogen, Carlsbad, CA, USA) and further purified using an RNeasy Mini Kit with RNase-Free DNase (both Qiagen, Valencia, CA, USA). cDNA was synthesized using either Superscript III reverse transcriptase or a SuperScript^® ^VILO™ cDNA Synthesis Kit (both Invitrogen) as described by the manufacturer. Bio-Rad iCycler and CFX96 machines were used for quantitative PCR employing Power SYBR^® ^Green (Applied Biosystems, Carlsbad, CA, USA) or SsoAdvanced SYBR^® ^Green Supermix (Bio-Rad, Hercules, CA, USA), respectively. The primer sequences used to amplify murine coding sequences of interest are presented in Table 1. Cycle threshold values were subjected to statistical analyses after normalization to glyceraldehyde-3-phosphate dehydrogenase.

**Table 1:** Primer sequences used to amplify murine coding sequences of interest

	Forward	Reverse
Dact2	5'-GGAGATGTGGGCACCGAGCG-3'	5'-GGCCAGTGCGGCTCGTAGTC-3'
DDR1	5'-GCCATGGTCACCTTGAAGCCAGC-3'	5'-CGATGAAGCCTCCCGGCTTTGTC-3'
Dsc2	5'-GCCCAGAGCTCCACCCTCGGA-3'	5'-ACACAGGCGCTTTTCTCGCGC-3'
eIF4GI	5'-CCGGTGGTGTTTAGCACGCCTC-3'	5'-CGGCTAGGGTAGAAGTGCTGCAG-3'
EpCAM	5'-AAGCCCGAAGGGGCGATCCA-3'	5'-GTGCCGTTGCACTGCTTGGC-3'
FAP	5'-CCAGGAGATCCACCTTTTCA-3'	5'-GTGGCAAGCATTTCCTCTTC-3'
GAPDH	5'-AGAACATCATCCCTGCATCC-3'	5'-CACATTGGGGGTAGGAACAC-3'
Gsc	5'-CGCCGAGCCAAGTGGAGACG-3'	5'-CCGGCGAGGCTTTTGAGGACG-3'
Mixl1	5'-CGCAAGCGCACGTCGTTCAG-3'	5'-GCGCTCCCGCAAGTGGATGT-3'
PyVmT	5'-TAAGAAGGCTACATGCGGATGGGT-3'	5'-GGCACCTGGCATCACATTTGTCTT-3'
Snai3	5'-CCACACGCTGCCCTGCATCT-3'	5'-GGGTGCGGATGTGACCCTGG-3'
SnoN	5'-GGCCACCAAGGCAGAGACAAATTC-3'	5'-GCTTGTGCCTCTCACTAAGCTGC-3'
Tmeff1	5'-GCCGAGTGTGACGAGGATGCG-3'	5'-AACTCCCGTCGGAAGCGCAC-3'
Wnt11	5'-TCCTGGGCTGGCAGGAGGAC-3'	5'-GACCAGGTCGGAGGACCGGG-3'

### Immunohistochemistry and immunofluorescence

*In ovo *tumors were harvested, fixed in 10% neutral buffered formalin, paraffin embedded, and sectioned. All immunohistochemistry and immunofluorescence involved blocking via incubation with 3% normal goat serum (Vector Laboratories). Immunohistochemistry for E-cadherin and phospho-Smad2 was completed by the Vanderbilt Translational Pathology Shared Resource. All immunofluorescence was performed using a standard pH 6 sodium citrate buffer. Immunofluorescence data were obtained using primary antibodies for vimentin (1:500, PCK-594P; Covance, Emeryville, CA, USA), α-smooth muscle actin (1:500, A2547; Sigma), E-cadherin (1:500, 610181; BD Transduction Laboratories, San Jose, CA, USA), cytokeratin 8/18 (1:500, 20R-CP004; Fitzgerald, Acton, MA, USA), ZO-1 (1:500, 61-7300; Zymed, San Francisco, CA, USA), p120 (1:400, 610133; BD Transduction Laboratories), and β-catenin (1:1,000, C2206; Sigma) by incubation overnight at 4°C. Corresponding Alexa Fluor^® ^secondary antibodies were used (1:1000; Invitrogen). Fluorescent imaging was completed on a Zeiss Axioplan upright widefield microscope (Thornwood, NY, USA).

### Immunoblotting

Protein lysate preparation and immunoblotting procedures were used as previously described [13]. Polyvinylidene difluoride membranes were blocked in 5% milk in Tris-buffered saline-Tween^® ^20 and incubated with primary antibody overnight at 4°C. The following primary antibodies were used: phospho-Smad2 (1:1,000, AB3849; Millipore, Billerica, MA, USA), TβRII (1:4,000, sc-400; Santa Cruz Biotechnology, Inc., Santa Cruz, CA, USA), Wnt11 (1:1,000, ab96730; Abcam, Cambridge, MA, USA), Tmeff1 (1:1,000, sc-98956; Santa Cruz Biotechnology, Inc.), Versican (1:1,000, AB1033; Millipore), and N-cadherin (1:2,500, 610920; BD Transduction Laboratories). Corresponding secondary horseradish peroxidase ImmunoPure^® ^antibodies were used (1:5,000; Pierce, Waltham, MA, USA). Chemiluminescence detection of protein was completed using Western Lightning^® ^ECL (Perkin-Elmer).

### Statistical analysis

All statistical analyses were reported using two-tailed unpaired *t *tests to determine significance (*P *< 0.05).

## Results

### Fibroblasts induce single cell/strand or collective migration of epithelia

To assess the inherent migratory differences between our murine MMTV-PyVmT TβRII KO or MMTV-PyVmT TβRII^fl/fl ^control mammary carcinoma cells, an *ex ovo *chicken embryo model system was employed. Initial grafting was of enhanced GFP-expressing murine MMTV-PyVmT mammary tumor epithelial cells, either TβRII KO or TβRII^fl/fl ^alone, which were allowed to form discernible, vascularized tumors for 3 days. Tumor-bearing animals were placed in an intravital imaging chamber and tumor cell motility was evaluated for up to 72 hours via time-lapse imaging. We observed a consistently larger tumor size of TβRII KO tumors compared with TβRII^fl/fl ^control tumors; however, both tumors presented no evidence of migration beyond the periphery of the primary tumor (see Figure S1 in Additional file 1). The lack of an inherent difference in migratory activity due to the presence or absence of TGF-β signaling in the epithelial cells confirmed that the previously published elevated lung metastasis observed in our TβRII KO mice was not due to enhanced cell-autonomous migratory capacity of TβRII KO epithelial cells alone. We therefore hypothesized that stromal influence on epithelial cells could critically alter the migration pattern of tumor epithelial cells.

**Figure 1:**
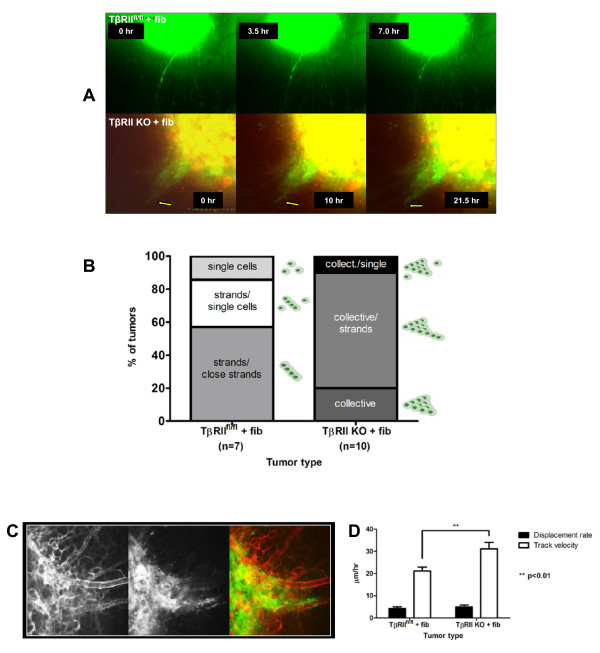
**Tumor-stromal interactions promoted either single cell or collective cell invasion**. Combinatorial xenografts of either enhanced GFP-labeled transforming growth factor-beta receptor II control (TβRII^fl/fl^) or transforming growth factor-beta receptor II knockout (TβRII KO) carcinoma cells with fibroblasts (fib) were grafted onto the chorioallantoic membrane and monitored via intravital imaging. **(A) **Top panel, single cell migration was exhibited in tumors that maintain epithelial transforming growth factor-beta (TGF-β) signaling. Only the epithelial channel is shown in order to visualize the single cell and strands displayed. Bottom panel, collective migration was observed in TβRII KO tumors (arrow). Both epithelial (green) and fibroblast (red) channels are overlayed. Fibroblasts guided both types of epithelial migration. **(B) **Migration types observed when comparing TβRII^fl/fl ^control and TβRII KO *ex ovo *tumors are quantified. **(C) **TβRII KO tumors migrated collectively along and around the vasculature, as shown by two-photon microscopy. Vasculature (left), epithelial (middle), and overlayed (right) panels are shown. The fibroblasts were unlabeled and therefore not shown. **(D) **Fibroblasts had enhanced velocity in the presence of TβRII KO epithelial cells compared with TβRII^fl/fl ^cells.

To best recapitulate tumor-stromal interactions of the tumor microenvironment, the TβRII^fl/fl ^and TβRII KO epithelial cells were combined with partial TβRII KO mammary fibroblasts *ex ovo *(hereafter, fibroblasts are grafted with epithelial cells in all tumors). Partial TβRII KO fibroblasts were used due to their ability to invoke more aggressive tumor behavior as compared with that of pure TβRII KO fibroblasts or TβRII competent fibroblasts [16]; however, each of these fibroblast cell lines were tested in our chicken embryo model and produced similar tumor migratory phenotypes as described below (data not shown). For the remainder of *in vivo *experimentation, only partial TβRII KO mammary fibroblasts were used. In both TβRII^fl/fl ^and TβRII KO tumors, the presence of fibroblasts caused epithelial migration away from the tumor periphery (Figure 1A; see Figure S1 in Additional file 1). In control TβRII^fl/fl ^tumors capable of TGF-β signaling, the tumor cells exhibited a strand and/or single cell migration (Figure 1A, B; see Additional file 2). Notably, collective migration was not observed in any TβRII^fl/fl ^tumors. In contrast, TβRII KO tumors exhibited primarily collective migration with occasional single cell or strand migration (Figure 1A, B; Additional file 3). In either tumor type, fibroblasts were always visible outside the tumor mass beyond the periphery of invading tumor cells, reaffirming the concept that stromal cells lead the way for subsequent tumor cell migration. This corroborates *in vitro *data indicating that fibroblasts enhanced the invasion of epithelial cells in a transwell assay (see Figure S2 in Additional file 1). The two migratory phenotypes observed *in vivo *were also affected by vascular influence in the tumor microenvironment. Migration appeared directional, as epithelial cells migrated along and around the vasculature (Figure 1C), perhaps due to migratory cues emanating from the vasculature or characteristics of the perivascular matrix.

Since the fibroblasts had a pronounced effect on tumor cell migration, a reciprocal effect of tumor cell influence on fibroblasts was investigated. No difference in displacement rate of fibroblasts from the tumor periphery was observed regardless of their combination with either TβRII^fl/fl ^or TβRII KO carcinoma cells; however, fibroblast velocity was increased by 50% in the presence of TβRII KO cells (Figure 1D). In this way, the TβRII KO epithelial cells, which possess an increased propensity for lung metastasis [12,13], responded to extrinsic stromal cues in a heightened manner and subsequently facilitated tumor-stromal communication. This reciprocity of tumor-stromal interactions in driving motility and invasion is consistent with previously observed interactions in the tumor microenvironment of other models [4,14,15,36].

### Cell migration mode can affect metastatic potential

**Figure 2:**
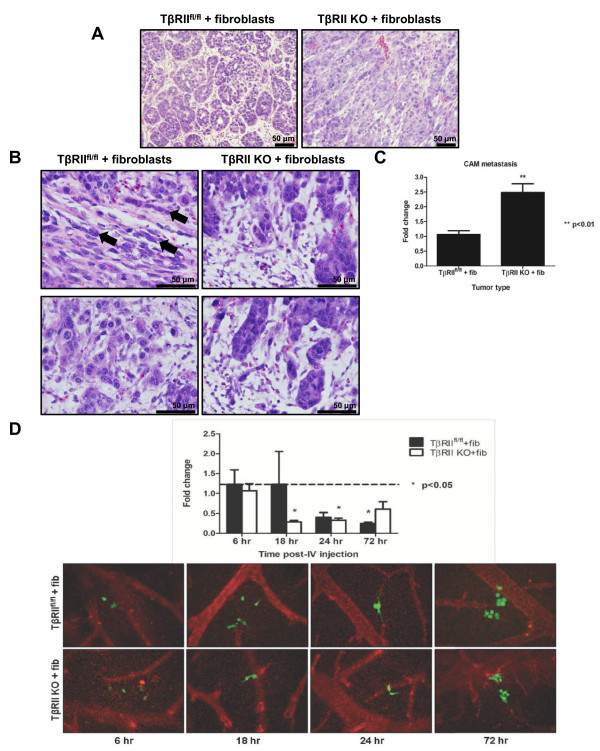
**Single cell and collective cell invasive aggregates demonstrated different metastatic potentials**. **(A) **H & E sections of *in ovo *tumors revealed overall tumor histology. **(B) **Evidence of strand filing (top left panel, arrows) and single cells (bottom left panel) were seen in H & E sections of transforming growth factor-beta receptor II control (TβRII^fl/fl^) tumors. Collective clusters were seen in transforming growth factor-beta receptor II knockout (TβRII KO) tumors. Images are representative of the tumor periphery and tumor-stromal boundaries. **(C) **Results from murine-specific *Alu *quantitative PCR found that collective aggregates of TβRII KO tumors achieved greater metastasis than single cells of TβRII^fl/fl ^tumors *in ovo*. CAM, chorioallantoic membrane; fib, fibroblasts. **(D) **TβRII KO epithelial cells possess a greater ability than TβRII^fl/fl ^cells to extravasate and survive post extravasation, quantified via an experimental metastasis assay and subsequent murine-specific *Alu *PCR (top graph). All timepoints and samples were compared with the 6-hour timepoint of TβRII^fl/fl ^cells and fibroblasts (dashed line). Representative images of epithelial cells (green) in relation to the lectin-labeled vasculature (red) were taken at all timepoints to confirm extravasation quantification and are shown beneath the graph (fibroblasts were unlabeled and therefore not shown). The 6-hour timepoint represented cells that arrested in the vasculature. Presence of carcinoma cells in the capillary bed, which is porous, was seen. At the 18-hour and 24-hour timepoints, proliferative capability of disseminated tumor cells was seen. This was evident in cells extravasating from the capillary bed, invading into areas of the CAM in close proximity to the vasculature, and exhibiting protrusive cellular processes. At the 72-hour timepoint, cohesive groups of cells with protrusive cellular processes were observed near vessels.

Histological evaluation of fixed tumor tissue was used to determine cellular morphology within the tumor. For this purpose, mammary carcinoma cells, either TβRII^fl/fl ^or TβRII KO, were combined with mammary fibroblasts and xenografted onto the CAM *in ovo*. Overall tumor histology revealed a well-differentiated, lobular morphology in TβRII^fl/fl ^control tumors; however, the TβRII KO tumors appeared less differentiated (Figure 2A). The tumor histology is not model dependent since CAM-xenografted tumors displayed similar morphology to that of the mouse models in which the grafted cells were generated [12,13]. Immunohistochemistry for phospho-Smad2 confirmed that TβRII^fl/fl ^tumors maintained TGF-β signaling in epithelial and stromal cells, while TβRII KO tumors lacked signaling in epithelia only (see Figure S3 in Additional file 1). At the cellular level, it is apparent that strand migration and numerous single epithelial cells were visible at the tumor-stromal interface and tumor edges of TβRII^fl/fl ^tumors (Figure 2B). In contrast, tumor cells at the tumor-stromal interface and tumor edges of TβRII KO tumors were visible as large clusters or cohorts. These findings corresponded with our observations during time-lapse imaging of cell migration (Figure 1A). One potentially confounding variable in our *in ovo *observations is the reproducibility with multiple xenografted cell lines. Using several carcinoma and fibroblast cell lines with the appropriate TβRII status, we therefore confirmed an identical pattern of single cell/strand migration (TβRII^fl/fl ^tumors) or collective migration (TβRII KO tumors) (see Figure S4 in Additional file 1).

Numerous publications have demonstrated that differential modes of cell migration can correlate with altered metastatic ability. In order to distinguish differential metastasis of TβRII^fl/fl ^or TβRII KO tumor cells, CAM distant from the primary tumor site was harvested from *in ovo *tumor-bearing animals. The amount of metastasis was then analyzed using murine-specific *Alu *PCR. Metastasis of collective aggregates in TβRII KO tumors was nearly 2.5-fold higher than that of TβRII^fl/fl ^tumors (Figure 2C). This data suggests that collective migration of cells lacking TGF-β signaling appeared to present a distinct advantage over single cell/strand migration of cells in stromal invasion. To further substantiate our metastatic findings, an *in ovo *experimental metastasis assay using murine-specific *Alu *PCR was performed. This assay detects the presence of epithelial cells in the CAM, initially upon vascular arrest and subsequently for extravasation and proliferative capability. TβRII^fl/fl ^carcinoma cells combined with fibroblasts maintained similar cell quantities upon vascular arrest and 18 hours post vasculature entry; however, the presence of these cells continued to decline over the course of the assay (Figure 2D). This decline was attributed to the inability of all cancer cells to survive in circulation and to the fact that fibroblast survival in circulation has not been well documented. In contrast to the behavior of the TβRII^fl/fl ^cells and fibroblasts, although TβRII KO carcinoma cells combined with fibroblasts resulted in a similar initial cell decline, there was a subsequent increase for the duration of the assay. This steady rise was attributed to better extravasation, survival, and colonization abilities of TβRII KO epithelia. This finding corroborates the CAM metastasis results, suggesting that the collective TβRII KO aggregates are better capable of metastasis (Figure 2C). In both cell combinations, it was also observed that the majority of extravasated cells were present in clusters near vasculature, with the TβRII KO epithelia forming more compact clusters (Figure 2D). The vascular proximity of colonizing cells supports our *in ovo *migratory results demonstrating directional vasculature migration (Figure 1C).

As confirmation of our extravasation results, an additional experimental metastasis assay was completed using carcinoma cells alone (see in Figure S5 Additional file 1). Although the presence of TβRII^fl/fl ^epithelial cells remained constant over the course of the assay, the TβRII KO epithelia were better able to extravasate and survive; however, neither the TβRII^fl/fl ^nor the TβRII KO epithelia had evidence of invasive cellular protrusions that were present when epithelial cells were combined with fibroblasts (Figure 2D; see Figure S5 in Additional file 1). Combining these two separate experimental metastasis assays suggests that the carcinoma cells may innately possess an extravasation ability that is enhanced by fibroblast presence. Investigation of intravasation capability, the initial step in metastatic dissemination, revealed no differences between the TβRII^fl/fl ^and TβRII KO epithelial cells (data not shown).

**Figure 3:**
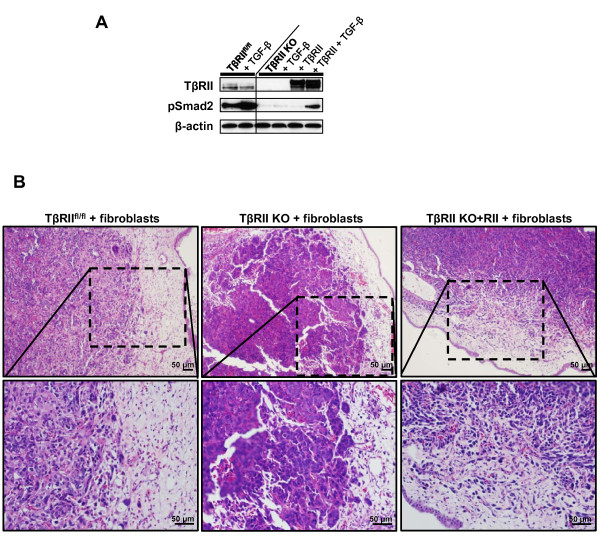
**Single cell migration is a transforming growth factor-beta receptor II -dependent event**. **(A) **Transforming growth factor-beta receptor II knockout (TβRII KO) cells used for xenografting were reconstituted with a functional human TβRII construct. Transforming growth factor-beta receptor II control (TβRII^fl/fl^) cells were used as a control for active transforming growth factor beta (TGF-β) signaling as assessed by phospho-Smad2 expression. A shorter exposure of the hTβRII blot was used for all TβRII KO lanes due to overexpression signal strength. **(B) **Reconstitution of active TGF-β signaling in TβRII KO epithelia recapitulated the single cell migratory phenotype observed in TβRII^fl/fl ^tumors.

To confirm that the observed migratory phenotypes were TβRII dependent, TβRII KO epithelial cells were reconstituted with functional TβRII (RII) [37] to regain responsiveness to TGF-β signaling (Figure 3A). *In ovo *xenografts of TβRII^fl/fl^, TβRII KO, or TβRII KO+RII were combined with fibroblasts, and migratory phenotype of the tumor cells was observed. Indeed, TβRII KO+RII epithelia showed evidence of single cell migration at the tumor periphery, thereby recapitulating the migratory phenotype observed in TβRII^fl/fl ^tumors (Figure 3B). These results substantiated the conclusion that single cell migration versus collective cell migration was a consequence of TβRII expression.

### Epithelia lacking TGF-β signaling maintain junctional protein localization at the tumor-stromal interface

**Figure 4:**
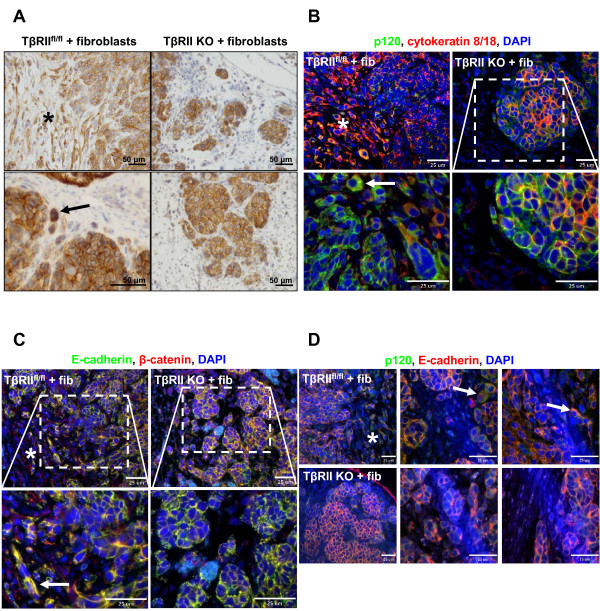
**Epithelial cell transforming growth factor-beta signaling disrupted maintenance of E-cadherin/p120/β-catenin membrane localization at adherens junctions**. All images were taken of *in ovo *tumors (asterisks, tumor-stromal regions; arrows, single cells with protein mislocalization). **(A) **Immunohistochemistry showed that E-cadherin was mislocalized in tumor-stromal regions in which single cells were found in transforming growth factor-beta receptor II control (TβRII^fl/fl^) tumors (top and bottom left panels). Collective clusters in the same regions exhibited E-cadherin membrane localization in transforming growth factor-beta receptor II knockout (TβRII KO) tumors (top and bottom right panels). **(B), (C), (D) **Immunofluorescence for p120, E-cadherin, and β-catenin revealed mislocalization of their expression in stromal areas of TβRII^fl/fl ^tumors but maintenance in TβRII KO tumors. Cytokeratin 8/18 was used as a marker for carcinoma cell identification. DAPI, 4',6-diamidino-2-phenylindole; fib, fibroblasts.

**Figure 5:**
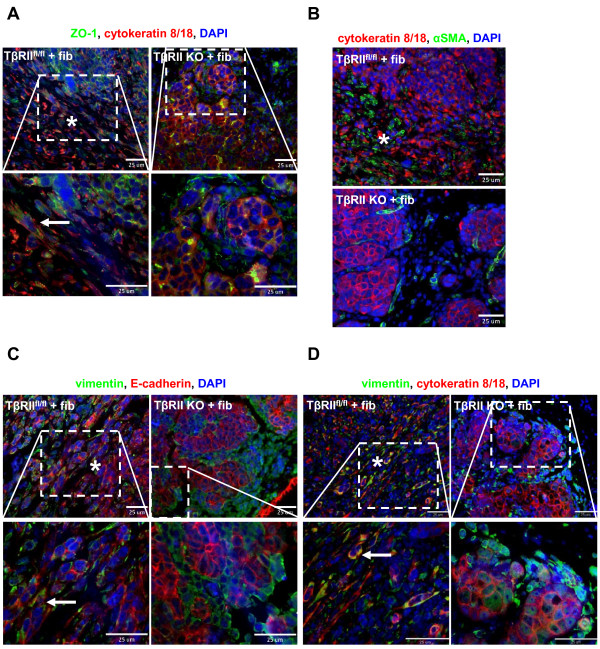
**Epithelial cell transforming growth factor-beta signaling disrupted tight junction protein localization while enhancing migratory expression**. All immunofluorescent images were taken of *in ovo *tumors (asterisks, tumor-stromal regions; arrows, single cells with protein mislocalization). Cytokeratin 8/18 was used as a marker for carcinoma cell identification. **(A) **ZO-1 was mislocalized in stromal areas of transforming growth factor-beta receptor II control (TβRII^fl/fl^) tumors but maintained in transforming growth factor-beta receptor II knockout (TβRII KO) tumors. **(B), (C), (D) **Increased expression of α-smooth muscle actin (α-SMA) and vimentin was seen in TβRII^fl/fl ^tumor cells located in tumor-stromal areas. Vimentin was expressed by fibroblasts immediately surrounding TβRII KO epithelial clusters. DAPI, 4',6-diamidino-2-phenylindole; fib, fibroblasts.

During development and tumorigenesis it is sometimes necessary for cells to maintain polarity and junctional adherence, albeit transiently [22,38]. This is important for effective forward migration of epithelial sheets during organ formation, as well as increased pressure of tumor epithelia to push against surrounding stroma during tumor proliferation. The divergent individual versus collective migratory phenotypes of TβRII^fl/fl ^and TβRII KO tumor cells observed in real-time imaging and in histological sections suggest that molecular distinctions responsible for cell-cell adhesion and migration are developed in response to TGF-β signaling. Indeed, immunohistochemical results indicated that E-cadherin expression was highly mislocalized in epithelia at the tumor-stromal interface of TβRII^fl/fl ^tumors (Figure 4A). Higher magnification revealed maintenance of E-cadherin membrane localization in multicellular lobular tumor structures but cytoplasmic localization or potential degradation in single epithelial cells. This contrasted with E-cadherin membrane localization in all collective clusters at the tumor-stromal interface of TβRII KO tumors. To further analyze junctional characteristics of the tumor types, cytokeratin 8/18 was used in immunofluorescence to distinguish epithelial cells from surrounding stromal cells. Results indicated that p120 and β-catenin were mislocalized in TβRII^fl/fl ^epithelia that possess TGF-β signaling, corresponding to the mislocalized E-cadherin evident in these tumors (Figure 4B, C, D). On the other hand, E-cadherin expression in clusters of TβRII KO tumors co-localized with both p120 and β-catenin expression at the membrane, suggesting maintenance of adherens junctions. Similarly, tight junctions also remained intact in TβRII KO tumors, as assessed by ZO-1 membrane localization, but were not maintained in TβRII^fl/fl ^tumors at the tumor-stromal interface (Figure 5A).

Since epithelial clusters in TβRII KO tumors maintained junctional protein expression, and epithelia of TβRII^fl/fl ^tumors appeared more mesenchymal, EMT-like markers were explored. As expected, epithelia in TβRII^fl/fl ^tumors, marked by cytokeratin 8/18, expressed α-smooth muscle actin and vimentin at the tumor-stromal interface and at the edges of lobular tumor structures (Figure 5B, C, D), confirming a mesenchymal phenotype. These observations are consistent with the idea that single cell migration may rely on classical mechanisms of EMT, such as loss of adherens and tight junctions and reorganization of actin stress fibers, to drive tumor cell invasion. Interestingly, all collective clusters in TβRII KO tumors were immediately surrounded by vimentin-positive adjacent fibroblasts. This finding corroborates our *ex ovo *findings (Figure 1A) and previous studies suggesting fibroblast-led migration of epithelial cells [17].

### Differing migration modes are associated with gene expression differences in *in ovo *tumors

**Figure 6:**
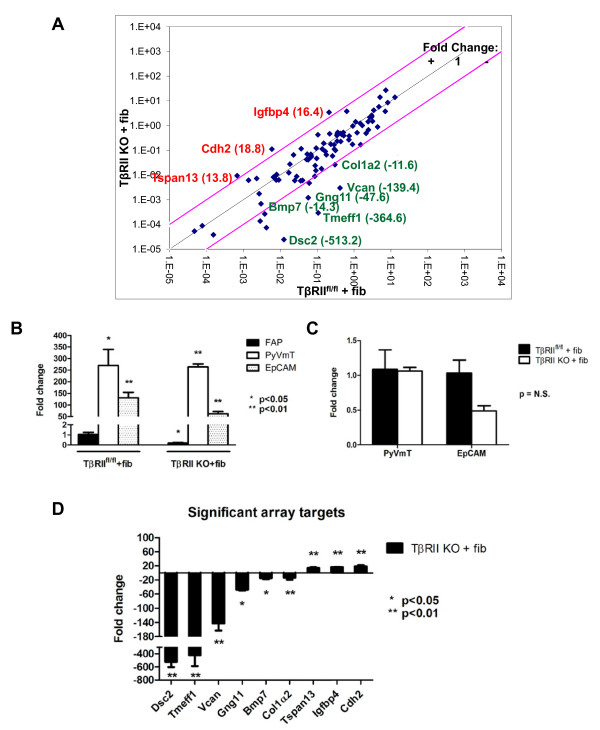
**Epithelial-to-mesenchymal transition gene expression changes were seen between tumors differing in invasive phenotype**. Gene expression changes detected on an epithelial-to-mesenchymal transition (EMT) quantitative PCR array were determined upon comparison of transforming growth factor-beta receptor II knockout (TβRII KO) isolated epithelia with transforming growth factor-beta receptor II control (TβRII^fl/fl^) isolated epithelia. **(A) **Identification of target genes was found by fold-change values. All highlighted genes were statistically significant (*P *< 0.05) and conform to the criteria of either 10-fold or greater upregulation or downregulation when comparing the TβRII KO laser capture microdissection (LCM) epithelia with the TβRII^fl/fl ^epithelia. **(B) **Epithelial purity of all LCM samples was confirmed when comparing PyVmT or EpCAM epithelial marker expression with that of the FAP fibroblast marker. All expression values were compared with FAP expression in the TβRII^fl/fl ^LCM sample. **(C) **Similar amounts of epithelia, as quantified by expression of PyVmT and EpCAM epithelial markers, were found in TβRII KO and TβRII^fl/fl ^LCM samples. **(D) **Array target gene expression (identified in (A)) of TβRII KO LCM samples, as compared with that of TβRII^fl/fl ^LCM samples, is shown with associated statistics. fib, fibroblasts.

**Figure 7:**
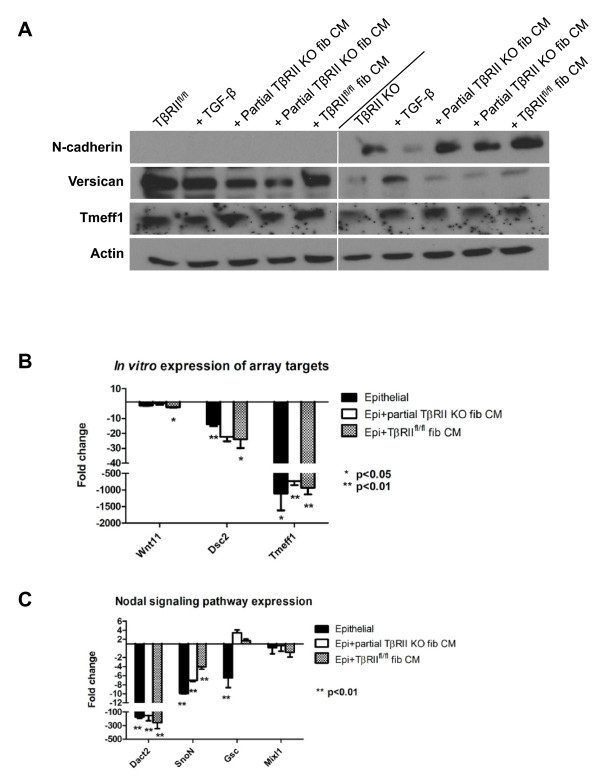
**Epithelial-to-mesenchymal transition gene expression changes were confirmed using cultured cells**. Cells used were the same as those xenografted onto the chorioallantoic membrane (see Figures 1 and 2). **(A) **Target gene validation was confirmed by immunoblotting. For either the transforming growth factor-beta receptor II control (TβRII^fl/fl^) cells or the transforming growth factor-beta receptor II knockout (TβRII KO) cells, the conditions were as follows: cells alone, cells treated with 1 ng/ml transforming growth factor beta (TGF-β) for 2.5 hours, cells treated with partial TβRII KO fibroblast conditioned media for 24 hours (two cell lines used), or cells treated with TβRII^fl/fl ^fibroblast conditioned media for 24 hours. Conditioned media treatment from partial TβRII KO and TβRII^fl/fl ^fibroblasts gave similar results. **(B) **Wnt11, Dsc2, and Tmeff1 expression in TβRII KO cells paralleled results seen in array results. For each condition (epithelial cells alone or fibroblast conditioned media treatment), all TβRII KO cell samples were respectively compared with TβRII^fl/fl ^cells. **(C) **Expression of Nodal signaling inhibitors (Dact2, SnoN) was downregulated but unaccompanied by significant expression increases of Nodal targets. For each condition (epithelial cells alone or fibroblast conditioned media treatment), all TβRII KO cell samples were respectively compared with TβRII^fl/fl ^cells. CM, conditioned media; fib, fibroblasts.

To identify gene expression changes that contribute to motility and invasion in response to loss of TGF-β signaling, we isolated tumor cells at the tumor-stromal interface using LCM on frozen *in ovo *tumor sections. For TβRII^fl/fl ^tumors, single migratory epithelial cells and epithelia lining the tumor-stromal interface were captured (see Figures S6 and S7 in Additional file 1). For TβRII KO tumors, migratory epithelial clusters in the stroma and epithelia lining the tumor-stromal interface were captured. Samples were then analyzed on an EMT quantitative PCR array (Figure 6A). Epithelial purity of the LCM samples was confirmed via PyVmT and EpCAM expression in comparison with FAP expression, markers of epithelia and fibroblasts, respectively (Figure 6B). It is important to note that the epithelial markers were similarly expressed in both TβRII^fl/fl ^and TβRII KO LCM samples, indicating the same quantity of epithelia in all LCM samples (Figure 6C). Using a 10-fold or greater upregulation or downregulation stringency for the EMT array, we identified upregulation of Cdh2, Igfbp4, and Tspan13, as well as downregulation of Col1α2, Bmp7, Wnt11, Gng11, Vcan, Tmeff1, and Dsc2 in TβRII KO epithelia compared with TβRII^fl/fl ^epithelia (Figure 6D). These target genes shared integral roles in cell-cell binding and growth factor signaling. Target expression was validated via immunoblot for N-cadherin, Vcan, and Tmeff1 (Figure 7A). Additionally, target expression of Wnt11, Tmeff1, and Dsc2 was confirmed via quantitative PCR on the cultured cell lines used for the *in vivo *assays (Figure 7B). Interestingly, the presence of fibroblast conditioned media induced similar gene expression changes to those seen by the LCM epithelia that were in the physical presence of fibroblasts. We also investigated some genes frequently associated with collective (DDR1, eIF4GI) [25,39] and mesenchymal migration (Snai3), but found no significant expression difference between our tumor types (see Figure S8 in Additional file 1).

One of the targets, Tmeff1, is a type I transmembrane receptor with signal transduction activity and is known to play a role in cancer progression signaling through induction of erbB4 tyrosine kinase receptor phosphorylation [40] and suppression of Nodal signaling. Tmeff1 inhibits Nodal signaling via binding to the Nodal co-receptor, Cripto [41], which is overexpressed in ~70 to 80% of invasive human breast cancer [42,43]. Increased expression of Tmeff1 has previously been shown as a direct result of Smad-dependent TGF-β signaling in the hair follicle [44]. Given that Tmeff1 is just one of several Nodal pathway inhibitors, we explored the expression of these other inhibitors. Dact2, which binds to activin type I receptors and targets them for lysosomal degradation, was ≥ 50-fold downregulated in TβRII KO epithelia across all *in vitro *conditions tested (Figure 7C). Downregulation of SnoN, an inhibitor of Nodal and TGF-β signaling, was also seen. Due to the observed downregulation of Nodal inhibitors, it might be inferred that activation of Nodal target genes would result. Surprisingly, only the Nodal target Gsc was upregulated in TβRII KO epithelia, while several other target genes (Mixl1, Nodal, Lefty 1/2, Ubr7, HESX1, Moap1, Cer1) were unaffected (Figure 7C; data not shown).

## Discussion

Patterns of carcinoma cell migration strikingly resemble those in development, organogenesis, tissue remodeling, and wound healing. During early embryogenesis EMT is frequently observed in gastrulation, while in late embryogenesis EMT is characteristic of neural crest migration [45,46]. Collective migration of epithelial sheets generates solidified epithelial barriers in organ development. Some of these sheets are led by tip cells that serve as a communication conduit to following cells in the cohort [23]. In mammary branching morphogenesis, the development and elongation of the mammary ductal tree involves collective invasion of terminal end buds [22,38]. Epithelial sheets and clusters maintain apicobasal polarity and cell-cell junctions. In these examples of cellular processes, cooperation is required between multiple cell populations, such as epithelial-stromal crosstalk. Evidence of both EMT and cohesive invasion can be found in our model of epithelial-stromal interactions within the tumor microenvironment. Fibroblasts were required for carcinoma cell invasion, suggesting a microenvironmental component of cellular communication. Our cohesively moving TβRII KO epithelia maintained adherens and tight junctional proteins necessary for cell-cell adhesion. The presence of vimentin-positive fibroblasts adjacent to these clusters further supports the concept of fibroblast-led epithelial invasion. Similar to EMT phenotypes seen in development, our TβRII^fl/fl ^tumors with competent TGF-β signaling express α-smooth muscle actin and vimentin and lose junctional polarity.

The predominant perception of TGF-β signaling in tumor migration has been that TGF-β induces single cell invasion, which is correlated with increased invasive and metastatic potential. This invasion has commonly been associated with epithelial cells undergoing EMT, through which they acquire mesenchymal characteristics of stromal cells and presumably become invasive. Yet recent evidence from *in vitro *studies finds a collective migration component of tumors [17]. There is histological evidence of chain or collective epithelial cell migration in human cancer. For many years, pathologists have identified cohorts of cells in stromal areas surrounding primary tumors [47]. In many instances, epithelial movement occurs within the epithelial-stromal interface of the tumor itself or at the tumor periphery. Consistent with current views, our work suggests that the presence of epithelial TGF-β signaling causes a single cell or strand migration. On the other hand, a lack of epithelial TGF-β signaling induces a collective tumor invasive front in the tumor areas prone to increased cell movement. Fibroblasts were able to induce these two varying patterns of migration. This suggests a pro-migratory effect provided by stromal fibroblasts that enables a cell-autonomous epithelial response dependent upon TGF-β signaling capability. A lack of TGF-β signaling has previously been implicated in collective migration, but this was shown through exogenous manipulation of the TGF-β pathway [48]. Our results, using genetic, cell-autonomous control of TGF-β signaling through expression of TβRII, specifically identified TGF-β as a critical factor involved in epithelial migration in the tumor microenvironment. The novelty of our findings also extended to the methodology by which we have achieved these results. Conventional *in vivo *imaging techniques afford minimal imaging length and significant viability issues inflicted on the animals used. The use of our cells in the CAM model enabled prolonged imaging and minimal embryo damage at each timepoint used for video capture.

A fluidity and plasticity between migration patterns is crucial to cancer progression. Beyond the characterization of tumor behavior at the primary site, the concept of mesenchymal-to-epithelial transition at secondary tumor sites has emerged [49-51]. In mesenchymal-to-epithelial transition, colonized metastases are histopathologically similar to the epithelial nature of the primary tumors from which they are derived [52,53]. These metastases possess polarity markers and a re-epithelialization that maintains junctional protein expression. This is evident in the movement of metastatic emboli, or clustered epithelia, which are a hallmark of inflammatory breast cancer [25]. Our work supports the epithelial nature of invasive cell movement. The collective aggregates observed in TβRII tumors were capable of greater CAM metastasis than were cells migrating singly or in strands that maintain TGF-β signaling. Additionally, our experimental metastasis assay results demonstrate that cells lacking TGF-β signaling possess an enhanced ability to extravasate, survive, and re-epithelialize at metastatic sites. The ability to colonize at distant sites, regardless of TβRII expression and cell quantity, is supporting evidence for an mesenchymal-to-epithelial transition. Since no difference in intravasation ability was found between tumors with and without TGF-β signaling, our results suggest that the extravasation and survival steps of the metastatic cascade may be where cells lacking TGF-β signaling have a distinct advantage in positively contributing to metastasis.

Our results begin to pinpoint a mechanism responsible for the clustered TβRII KO epithelial invasion versus the single cell or strand migration of TGF-β-competent epithelia. Tmeff1 is a crucial inhibitor of the Nodal signaling pathway, which is responsible for many EMT-related effects. It is therefore noteworthy that our TβRII KO epithelia significantly downregulated Tmeff1 yet maintained a clustered aggregate formation during invasion. We showed that other Nodal signaling pathway inhibitors were also downregulated. Our results allude to a significant overlap between TGF-β and Nodal signaling pathways as a consequence of TβRII loss. Given that Tmeff1 contains Smad-binding elements in its promoter and has been shown to be activated in Smad-dependent TGF-β signaling in the hair follicle [44], it is likely also a TGF-β target in the mammary gland, a question further being pursued. Tmeff1 may also be regulated by a fibroblast-secreted factor in the tumor microenvironment. Our results using fibroblast conditioned media suggest that the physical presence of fibroblasts may not be necessary to induce gene expression changes responsible for migration patterning. This corroborates previously published studies implicating the role of fibroblast-secreted factors in tumor cell proliferation and motility [16,54].

Our findings illustrate a critical role for TGF-β signaling in the regulation of tumor microenvironmental interactions. Epithelial-stromal signaling deserves further study as a prominent driver of invasive and metastatic progression. The presence of fibroblasts induces specific carcinoma cell migration patterning dependent upon TGF-β competency. Further characterization of single cell migration versus collective cell migration is needed in tumor analysis in order to better understand the contribution of each to tumor progression. Upon further investigation, it is the hope that specific patterns of tumor invasiveness can be targeted as recourse for breast cancer treatment.

## Conclusion

Our findings implicate a role for TGF-β signaling in the regulation of epithelial migration patterning in the tumor microenvironment. We have shown that lack of epithelial TGF-β signaling induces a collective invasion of epithelia in the presence of stromal influence, while the presence of TGF-β signaling induces a single cell or strand migration. While stromal cells are needed for induction of epithelial invasion, we have shown cell-autonomous migration pattern response to this stimulus. The altered expression of Tmeff1 was also identified as a consequence of these migration differences. Our results are important in identifying invasive cellular behavior that can be targeted in hopes of preventing the metastatic spread of breast cancer.

## Abbreviations

CAM: chorioallantoic membrane of a chicken embryo; EMT: epithelial-to-mesenchymal transition; GFP: green fluorescent protein; H & E: hematoxylin and eosin; LCM: laser capture microdissection; MMTV: mouse mammary tumor virus; PyVmT: polyoma virus middle T antigen; PCR: polymerase chain reaction; RII: construct of functional TβRII; TβRII: type II transforming growth factor-beta receptor; TβRII^fl/fl^: transforming growth factor-beta receptor II control; TβRII KO: transforming growth factor-beta receptor II knockout; TGF-β: transforming growth factor beta.

## Competing interests

The authors declare that they have no competing interests.

## Authors' contributions

LAM was involved in study conception, all experiments/data analyses, and drafting of the manuscript. TDP was instrumental in assisting with all CAM experiments and had a significant role in data analysis and interpretation. WJA aided in technical troubleshooting for CAM experiments, as well as with computerized analysis of results. AN grafted cells for *ex ovo *assays and was involved in data analysis. AC and MA assisted in *in vitro *cell maintenance and experiment coordination. MWP provided critical insight about the study design and experimental interpretation. AEG performed immunoblotting for TβRII reconstitution experiments and was involved in data interpretation. AZ and HLM were primary contributors to study conception, design, and experimental implementation. All authors read and approved the final manuscript.

## Supplementary Material

Additional file 1 **Figure S1 showing that fibroblasts caused increased tumor growth of both TβRII^fl/fl ^and TβRII KO tumors (top panels, epithelial cells alone; bottom panels, epithelia and fibroblasts combined)**. Epithelial cells in green, fibroblasts overlayed in red. Figure S2 showing that fibroblasts enhanced invasion of carcinoma cells through Matrigel-coated (BD Biosciences) transwells after 6 hours. Carcinoma cells were permitted to invade through Matrigel alone. Carcinoma cells were also allowed to invade through Matrigel that had a bottom fibroblast coating used to assess tumor-stromal interactions. Figure S3 showing that TβRII^fl/fl ^tumors maintain epithelial and stromal TGF-β signaling as indicated through phospho-Smad2 expression, while TβRII KO tumors maintain TGF-β signaling only in the partial TβRII KO fibroblasts. Figure S4 showing that additional TβRII^fl/fl ^and TβRII KO epithelial cell lines were combined with fibroblasts to confirm similar *in ovo *histology as that observed in tumors detailed in this manuscript. Overall histology (top panels) and single cell (bottom left panel) or collective migration (bottom right panel) are shown. Figure S5 showing that TβRII KO epithelial cells possess a greater ability than do TβRII^fl/fl ^cells to extravasate and survive post extravasation. This was quantified via an experimental metastasis assay and subsequent murine-specific *Alu *PCR (top graph). All timepoints and samples were compared with the 6-hour timepoint of the TβRII^fl/fl ^cells (dashed line). Representative images of epithelial cells (green) in relation to the lectin-labeled vasculature (red) were taken at all timepoints to confirm extravasation quantification and are shown beneath the graph. The 6-hour timepoint represented cells that arrested in the vasculature. Presence of carcinoma cells in the capillary bed, which is porous, was seen. At the 18-hour and 24-hour timepoints, proliferative capability of disseminated tumor cells was seen. This was evident in cells extravasating from the capillary bed and invading into areas of the CAM in close proximity to the vasculature. Figure S6 showing representative H & E sections of *in ovo *tumors. Circled and highlighted areas of the tumor indicate which carcinoma cells were chosen for isolation by laser capture microdissection. Figure S7 showing sections of *in ovo *tumors prior to (left panels) and after (middle panels) LCM. The material obtained on the LCM cap is also shown (right panels). Figure S8 showing that no significant differences in DDR1, Snai3, or eIF4GI expression between TβRII^fl/fl ^and TβRII KO LCM tumor epithelia were seen via quantitative PCR analysis. Only expression fold-changes of TβRII KO LCM epithelia, as compared with TβRII^fl/fl ^LCM epithelia, are shown. Click here for file

Additional file 2 **A representative time-lapse movie of *ex ovo *TβRII^fl/fl ^control tumor migration monitored through intravital imaging**. Carcinoma cells and fibroblasts were xenografted together to form the tumor, but only the carcinoma cell channel is shown. Single cell and strand migration were observed. Click here for file

Additional file 3 **A representative time-lapse movie of *ex ovo *TβRII KO tumor migration monitored through intravital imaging**. Carcinoma cells and fibroblasts were xenografted together to form the tumor, but only the carcinoma cell channel is shown. A predominant peak of collective migration was observed along with a few singly migrating cells. Click here for file
