# Supplementary material for: Lack of transforming growth factor-β signaling promotes collective cancer cell invasion through tumor-stromal crosstalk
Source: Breast Cancer Res. 2012 Jul 2;14(4):R98. doi: 10.1186/bcr3217 (PMC3680921; doi:10.1186/bcr3217)
Supplement: Additional file 1 — Figure S1 showing that fibroblasts caused increased tumor growth of both TβRIIfl/fl and TβRII KO tumors (top panels, epithelial cells alone; bottom panels, epithelia and fibroblasts combined). Epithelial cells in green, fibroblasts overlayed in red. Figure S2 showing that fibroblasts enhanced invasion of carcinoma cells through Matrigel-coated (BD Biosciences) transwells after 6 hours. Carcinoma cells were permitted to invade through Matrigel alone. Carcinoma cells were also allowed to invade through Matrigel that had a bottom fibroblast coating used to assess tumor-stromal interactions. Figure S3 showing that TβRIIfl/fl tumors maintain epithelial and stromal TGF-β signaling as indicated through phospho-Smad2 expression, while TβRII KO tumors maintain TGF-β signaling only in the partial TβRII KO fibroblasts. Figure S4 showing that additional TβRIIfl/fl and TβRII KO epithelial cell lines were combined with fibroblasts to confirm similar in ovo histology as that observed in tumors detailed in this manuscript. Overall histology (top panels) and single cell (bottom left panel) or collective migration (bottom right panel) are shown. Figure S5 showing that TβRII KO epithelial cells possess a greater ability than do TβRIIfl/fl cells to extravasate and survive post extravasation. This was quantified via an experimental metastasis assay and subsequent murine-specific Alu PCR (top graph). All timepoints and samples were compared with the 6-hour timepoint of the TβRIIfl/fl cells (dashed line). Representative images of epithelial cells (green) in relation to the lectin-labeled vasculature (red) were taken at all timepoints to confirm extravasation quantification and are shown beneath the graph. The 6-hour timepoint represented cells that arrested in the vasculature. Presence of carcinoma cells in the capillary bed, which is porous, was seen. At the 18-hour and 24-hour timepoints, proliferative capability of disseminated tumor cells was seen. This was evident in cells extravasat [file bcr3217-S1.PDF]

S1

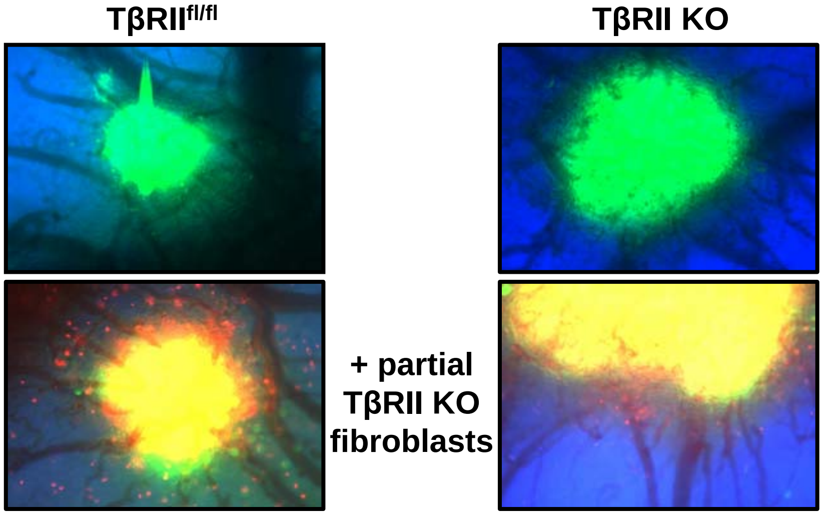
TβRIIfl/fl
TβRII KO
+ partial
TβRII KO
fibroblasts

S2

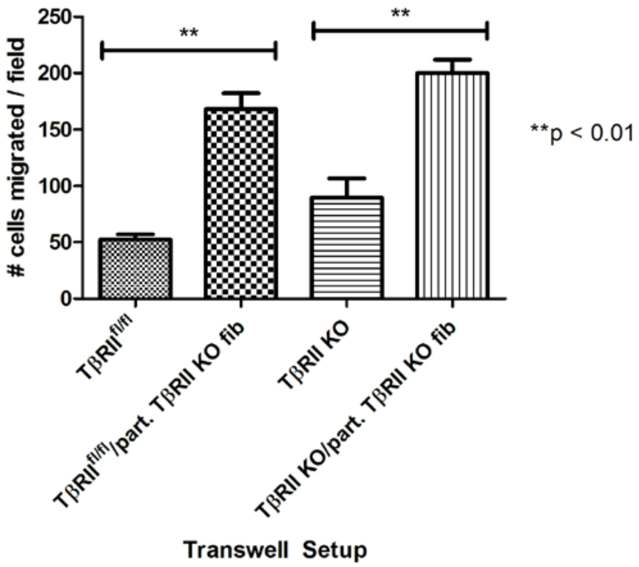
250
200
150
100
50
0
cells migrated / field
**
**
**p < 0.01
TβRIIfl/fl
TβRIIfl/fl/part. TβRII KO fib
TβRII KO
TβRII KO/part. TβRII KO fib
Transwell Setup

S3

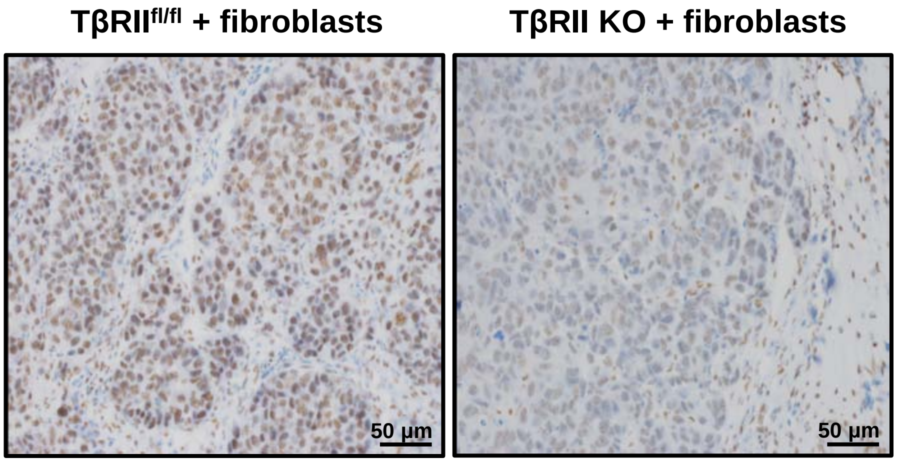
TβRIIfl/fl + fibroblasts
TβRII KO + fibroblasts
50 μm
50 μm

S4

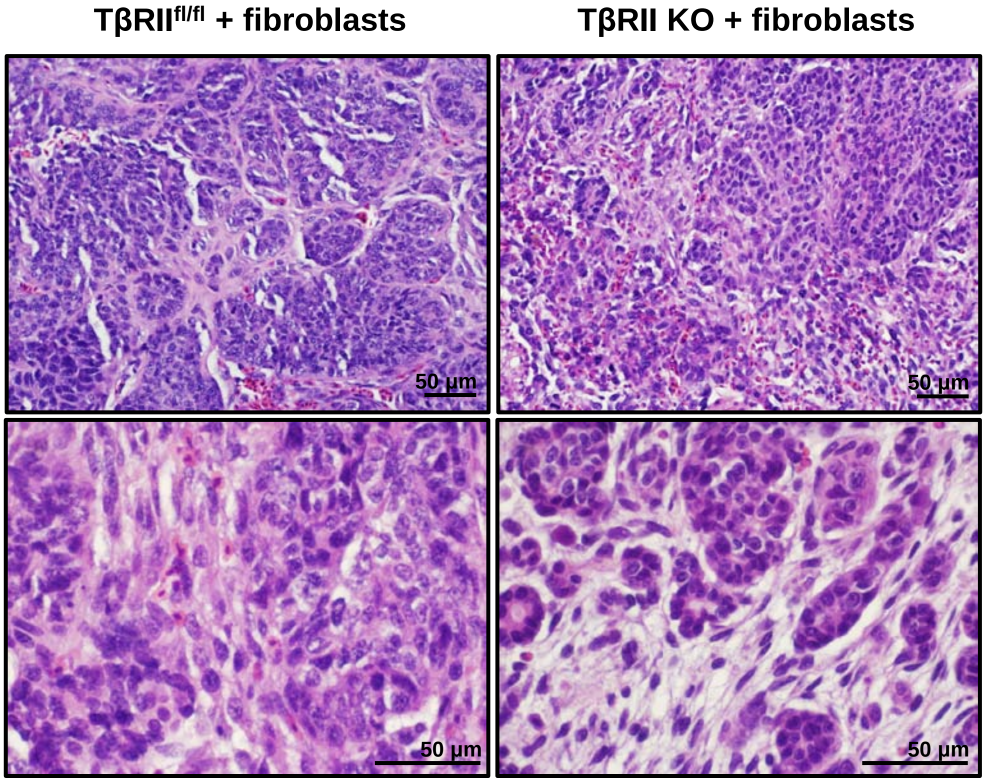
TβRIIfl/fl + fibroblasts
TβRII KO + fibroblasts
50 μm
50 μm
50 μm
50 μm

S5

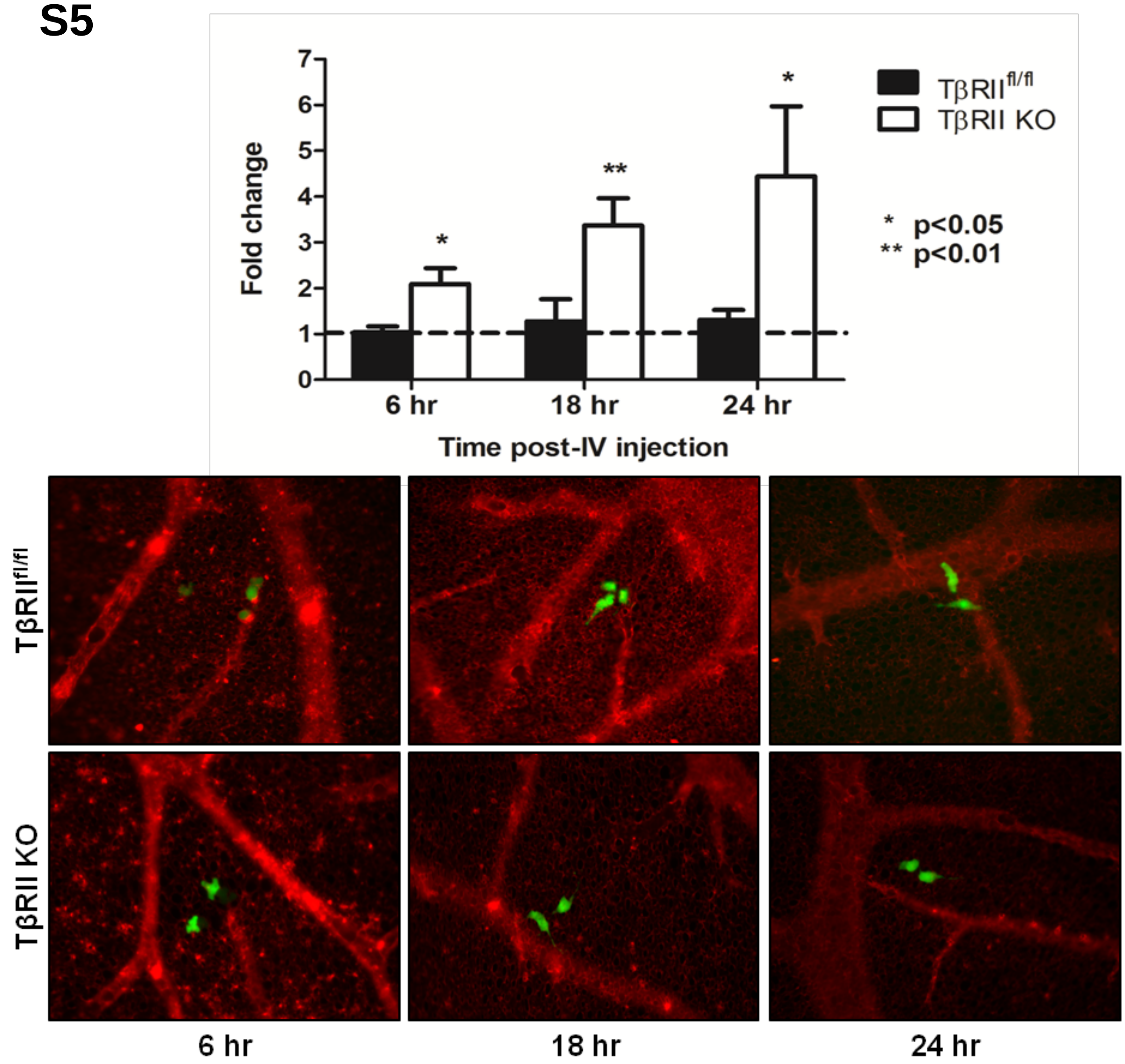
Fold change
0
1
2
3
4
5
6
7
*
**
*
6 hr
18 hr
24 hr
Time post-IV injection
TβRIIfl/fl
TβRII KO
* p<0.05
** p<0.01
TβRIIfl/fl
TβRII KO
6 hr
18 hr
24 hr

S6

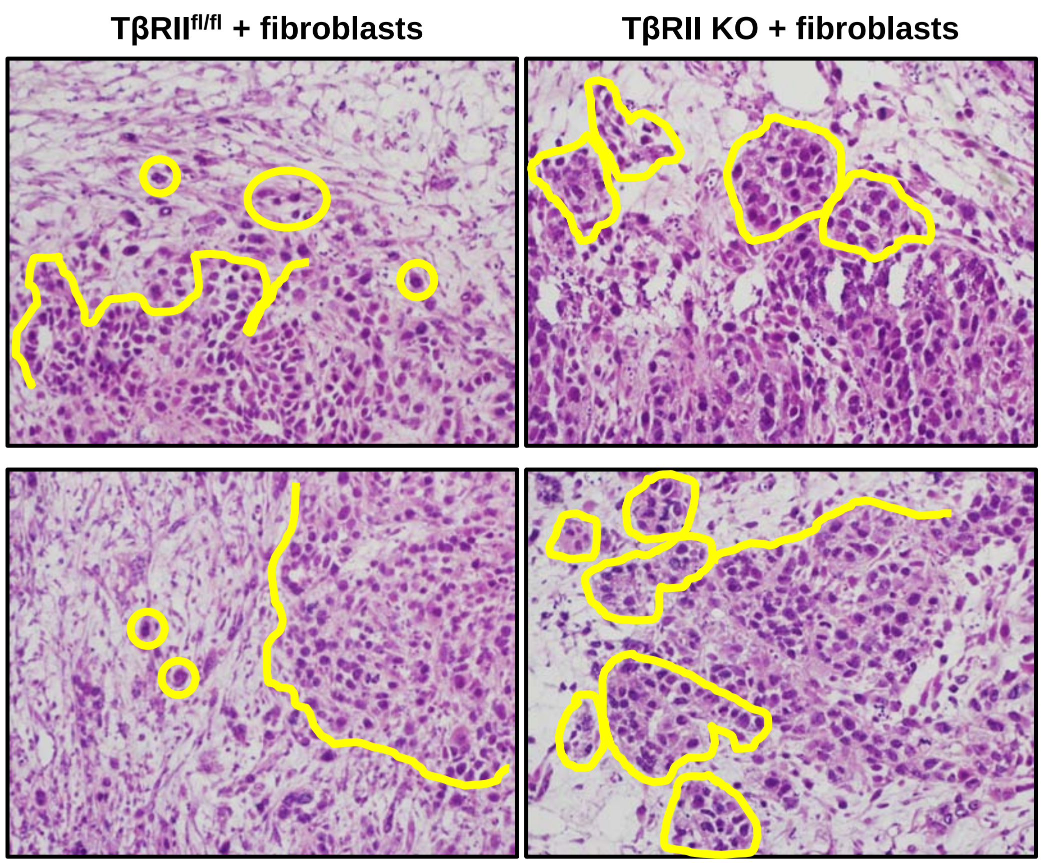

TβRII^fl/fl + fibroblasts
TβRII KO + fibroblasts

S7

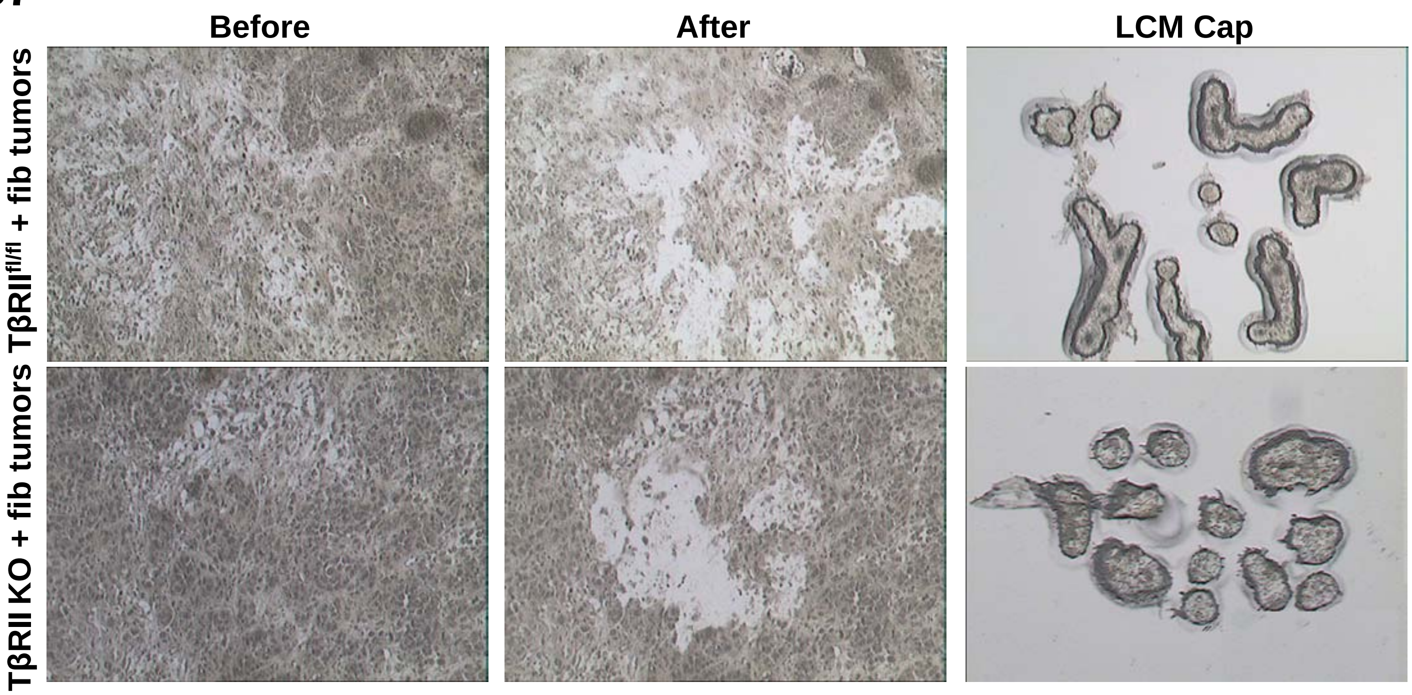

Before
After
LCM Cap
TβRII^fl/fl + fib tumors
TβRII KO + fib tumors

S8

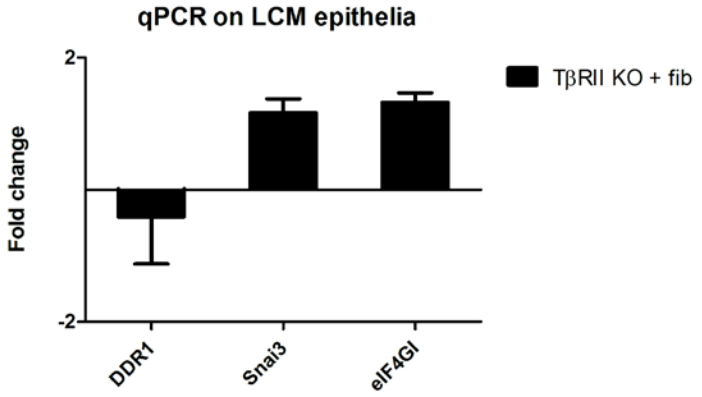

qPCR on LCM epithelia
TβRII KO + fib
Fold change
2
-2
DDR1
Snai3
eIF4GI
